# Barriers to Decolonizing Global Health: Identification of Research Challenges Facing Investigators Residing in Low- and Middle-Income Countries

**DOI:** 10.9745/GHSP-D-23-00269

**Published:** 2024-02-28

**Authors:** Nana Anyimadua Anane-Binfoh, Katelyn E. Flaherty, Ahmed N. Zakariah, Eric J. Nelson, Torben K. Becker, Taiba Jibril Afaa

**Affiliations:** a Department of Child Health, Korle Bu Teaching Hospital, Accra, Ghana.; b Department of Environmental and Global Health, College of Public Health and Health Professions, University of Florida, Gainesville, FL, USA.; c Section of Global Health, Department of Emergency Medicine, College of Medicine, University of Florida, Gainesville, FL, USA.; d Ghana National Ambulance Service, Accra, Ghana.; e Department of Pediatrics, College of Medicine, University of Florida, Gainesville, FL, USA.; f Center for African Studies, University of Florida, Gainesville, FL, USA.; g Department of Child Health, University of Ghana Medical School, College of Health Sciences, University of Ghana, Accra, Ghana.

## Abstract

The practice of global health is plagued by power structures favoring high-income countries. Efforts to decolonize global health must consider the systemic limitations that LMIC investigators face at local, national, and international levels.

## Key Messages

- Local limitations on protected time for research, ethical review, technology, and training threaten the productivity and development of investigators in low- and middle-income countries.
- National governments of many low- and middle-income countries underprioritize research or thwart its progress through political repression or instability.
- At the international level, investigators in low- and middle-income countries must compete with advantaged investigators from high-income nations for funding and publications.

## INTRODUCTION

The field of global health aims to leverage global partnerships to investigate issues transcending local boundaries.^1^ It acknowledges the importance of forming collaborative teams with diverse knowledge and experience to combat health disparities.^2^ However, in practice, global health is dominated by high-income countries (HICs). Most global health centers, global health conferences, and corresponding authors in global health journals are located in HICs.^3^^–^^5^ Though income is an imperfect classifier of countries, the dominance of HICs hints at the colonial legacy plaguing global health.

Global health’s colonial legacy stems partly from the concept of “tropical medicine,” which was born from the necessity to understand diseases in areas of the world occupied by European colonial powers with economic, political, or military agendas. Today, global health remains plagued by power structures based upon colonial legacies of inequity and agendas mired in priorities set by HICs. These structures and agendas drive HICs to engage within low- and middle-income country (LMIC) health systems without prioritizing partnerships with LMIC investigators.^6^ This practice threatens the global health promise of equity and justice and decreases the LMIC relevance and, thus, the quality of global health projects.^7^

There are mounting efforts to decolonize global health and work toward a future where investigators from LMICs and HICs engage in equitable partnerships.^8^ To that end, the University of Washington developed a Decolonizing Global Health Toolkit to help research teams acknowledge and dismantle power dynamics rooted in colonial legacies.^9^ The toolkit guides teams in assessing power structures using the Decolonization Power Structure Assessment Framework before and during study implementation (Box). The framework calls for researchers to (1) identify project roles, (2) describe existing power dynamics, (3) explore barriers to equitable partnership, and (4) take action to overcome the barriers.

BOX Applying the Decolonization Power Structure Assessment Framework to MotoMeds Telemedicine and Medication Delivery Service in Ghana

**Roles:** Our leadership team consists of a U.S.-based principal investigator (PI) along with Ghanaian and U.S.-based co-investigators. All U.S.-based investigators and 1 Ghanaian co-investigator were involved with the project before funding. All investigators were involved in pre-implementation project design. Throughout implementation (ongoing), U.S.-based and Ghanaian senior investigators provide oversight. U.S.-based and Ghanaian trainee investigators provide nightly operational and clinical advice, respectively. U.S.-based investigators do not receive additional income for their engagement in MotoMeds; however, their institution is compensated for indirect costs. Ghanaian investigators are directly paid for their work with MotoMeds. All investigators derive educational benefits and professional recognition for their roles in MotoMeds.

**Power dynamics:** Although all investigators contribute to project decision-making, the PI holds ultimate decision-making power. He also has the most direct line of contact with the study funder, the U.S. Agency for International Development (USAID), and will serve as the corresponding author on future MotoMeds publications. The U.S.-based trainee investigator will lead manuscript preparation and the authorship line under the PI’s mentorship. All investigators will edit the manuscript and be listed as authors.

**Barriers to equitable partnership:**

- **Local**: The U.S.-based trainee investigator has more protected research time and technical writing training than the Ghana-based trainee investigator and thus is better positioned to lead manuscript preparation.
- **National**: The Ghanaian government has not allocated the Ghana National Ambulance Service sufficient funds to absorb MotoMeds into its operations; thus, MotoMeds implementation remains reliant on USAID funding.
- **International**: USAID tends to fund projects with PIs based in the United States. High-impact journals in global health may be more apt to publish a MotoMeds article with a corresponding author from a HIC.

**Actions:**

- We may overcome local barriers by providing the Ghanaian trainee investigator with the necessary support (i.e., compensated time and training in technical writing) to co-lead manuscript preparation.
- We have little influence over national challenges; however, we may address international barriers by including a Ghanaian Co-PI on future MotoMeds grants and a Ghanaian corresponding author on MotoMeds publications.
- We also should support USAID in their stated intent to provide more funding to projects with PIs in low- and middle-income countries.

In this commentary, we seek to expand on the Decolonization Power Structure Assessment Framework by identifying and discussing specific barriers facing LMIC investigators. We organize our discussion from local to international scales to emphasize how small-scale challenges contribute to global barriers (Figure). Barriers at multiple levels must be acknowledged and addressed to achieve global health equity.

**FIGURE:**
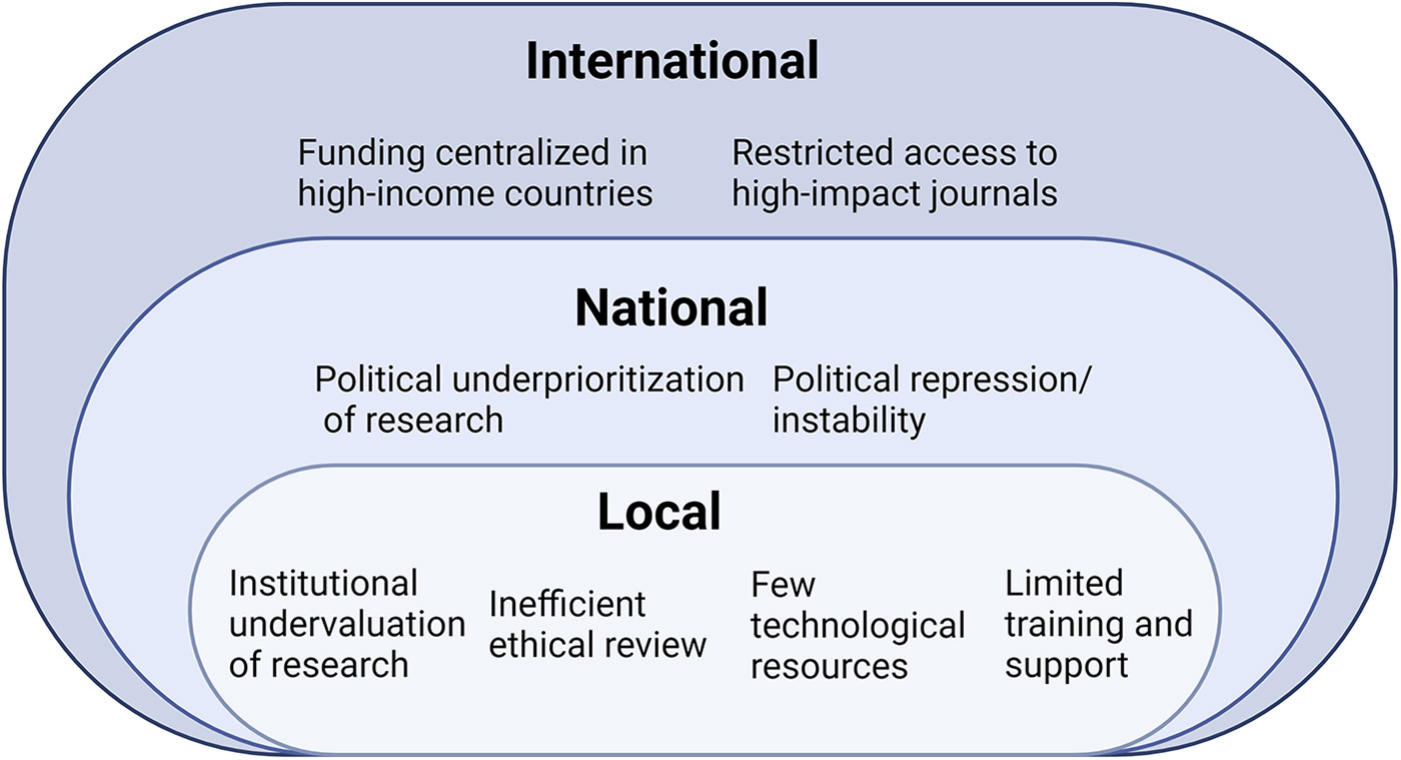
Research Challenges Facing Global Health Investigators in Low- and Middle-Income Countries^a^ ^a^ Barriers exist on multiple interdependent scales.

We approach this discussion aware of the strengths and limitations of our authorship team, which consists of investigators from Ghana, Europe, and the United States with a history of collaborating on health research projects in Ghana. We are diverse in race, gender, and ethnicity, as well as training and experience level. Our team includes individuals ranging from trainees to senior researchers trained in various health and research disciplines (pediatrics, emergency care, microbiology, and implementation science). However, we are homogenous in that we are all clinicians who primarily conduct clinical research centered on patient outcomes. We are further limited in that our LMIC authorship is from a single country: Ghana. Given these limitations, we draw upon global literature to anchor our reflections.

## LOCAL BARRIERS

Local challenges threaten LMIC investigators’ productivity and development, thus placing them at a baseline disadvantage compared to their HIC counterparts.

### Institutional Undervaluation of Research

Universities in LMICs serve as educational bodies with greater emphasis on teaching than research.^10^ Protected research time is rare, and research-based promotion opportunities are ambiguous and limited.^11^ Investigators are expected to use personal funds for research activities, such as conferences; however, financial compensation for research is poor.^12^ Many investigators must support themselves through heavy teaching loads or secondary employment in private sectors, thus limiting their time for research.^10^^,^^13^ We, the Ghanaian authors, are among the many LMIC investigators who conduct our research on our own time during nights and weekends.

### Inefficient Ethical Review

Another factor limiting LMIC investigators’ productivity is the ethical review process. Health research ethics are critical in ensuring the rights and safety of all research participants. As such, institutions possess research ethics committees tasked to review, approve, and oversee research involving human subjects. In LMICs, many ethical review committees suffer from long turnaround times due to understaffing and underfunding.^14^ Committee productivity is further compromised by limited staff training and competing ethical and scientific demands.^15^^,^^16^ A 2021 study of African countries found that 80% employ combined ethical and scientific review committees, which some argue delays research clearance and threatens committee expertise.^16^ In our work, we have experienced year-long delays in ethical review due to committee strikes and subsequent study backlogs. Our project launch dates are frequently postponed.

### Few Technological Resources

Novel health technologies are rapidly emerging. Innovations in imaging, diagnostics, digital health, and robotics provide investigators with more technological resources for studying health than ever before. Unfortunately, the price point associated with these cutting-edge technological resources is such that LMICs rarely can afford to benefit from such innovations.^17^ LMICs with access to these cutting-edge technologies are limited in terms of technical support to maintain these technologies, which is frequently based in HICs.^18^ As such, LMIC investigators are forced to rely on less sophisticated research tools, which often take longer to use or provide less information than their digital counterparts.^19^ For example, we have been forced to collect data on paper forms instead of digital applications due to our lack of tablets and poor Internet connectivity.

### Limited Training and Support

In response to local limitations on research productivity, many LMIC investigators opt to emigrate to HICs in search of better pay and better conditions.^20^ One of the many adverse effects of the emigration of professionals (“brain drain”) is a lack of qualified faculty to offer learning opportunities in LMICs.^10^^,^^21^ The absence of experienced faculty limits student training in various disciplines, including technical writing and English as a foreign language, both of which are necessary to gain research funding and visibility.^17^ Further, research mentorship programs are few during schooling and after graduation.^22^ Young researchers are immediately expected to obtain independent funding rather than hone their research capabilities under the guidance of experienced investigators.^23^ As such, we LMIC investigators rely on unofficial mentorship from senior investigators who recall facing training and support barriers earlier in their careers.

## NATIONAL BARRIERS

National challenges threaten the self-sufficiency of LMICs, thus forcing LMIC investigators to face global competition for foreign support.

### Political Underprioritization of Research

Political priorities determine government resource allocation. In 2007, member nations of the African Union committed to investing at least 1% of the gross domestic product in research and development. Countries such as Egypt, Kenya, and South Africa are near the 1% target; however, no member of the African Union has yet managed to fulfill the commitment.^24^ Governmental investment in research and development is particularly low in sub-Saharan Africa, where the average proportion of the gross domestic product dedicated to research and development remains below 0.5%.^24^ These values speak to the research pessimism in LMICs; many bureaucrats perceive research expenditures to be largely fruitless.^25^ In Ghana, the government seems to allocate resources to development projects in which short-term progress can be appreciated, such as the construction of the National Cathedral and Marine Drive, instead of investing in research, which offers delayed gratification.

### Political Repression/Instability

In some LMICs, national governments not only underprioritize research but thwart its progress. In the setting of political repression and persecution, investigators’ abilities to collect and publish information freely may be limited.^26^ Imposed restrictions may, in turn, threaten the integrity of the research by introducing bias.^27^ In the setting of political instability, the turnover of government leaders and parties fragments research initiatives.^13^ Instability that escalates to violence may damage research infrastructure and inhibit investigators’ safety at work.^27^

## INTERNATIONAL BARRIERS

On a global scale, LMIC investigators compete with HIC investigators for opportunities based in HICs that are awarded based on standards set by HICs.

### Funding Centralized in High-Income Countries

In 2000, the Global Forum for Health Research presented the “10/90 gap”: 10% of the global spending on health research is dedicated to conditions accounting for 90% of the global disease burden.^28^ The Forum called to address the misallocation of funding by empowering and enabling LMIC investigators to study their local conditions.^28^ However, investigators in LMICs continue to struggle to obtain research funding as most global health funders are based in HICs.^10^^,^^17^ HIC-based funders, including those with a global health focus, typically fund projects with principal investigators in their own countries. Investigators in the United States receive 80% of U.S. Agency for International Development contracts and 70% of Fogarty grants. Projects in the United Kingdom receive 73% of Wellcome Trust funding.^29^ The literature comparing grant submission and rejection rates between HIC and LMIC investigators is limited; however, per our experience, we feel that grants with principal investigators in LMICs are rejected more frequently than those submitted by investigators in HICs. Reasons for this may include distrust of investigators from LMICs or a lack of understanding of LMIC-oriented projects described by LMIC investigators.

### Restricted Access to High-Impact Journals

The literature comparing submission and rejection rates of manuscripts authored by HIC and LMIC investigators is similarly limited. We feel that this gap in the literature may mask journal biases against LMIC authorship. In our experience, investigators from LMICs often struggle to obtain article acceptance in high-impact, international journals; thus, they are forced to publish in local journals that frequently are unindexed or not digitized, resulting in low coverage. A review of authorship in high-impact global health journals from 2014 to 2016 found that less than a quarter of corresponding authors were affiliated with LMICs.^4^ Correspondingly, a study of authorship trends in *Lancet Global Health* found that 82% of articles from 2013 to 2017 focused on LMICs, yet only 35% of authors were affiliated with LMICs.^30^ We feel that the underrepresentation of LMIC authors in global health is driven by not only low submission rates but also concerns over the quality of research/writing conducted by LMIC investigators. As such, publishing challenges relate back to local challenges limiting research productivity and technical training.

## CONCLUSION

As a collaborative team of authors from LMIC and HIC settings, we have identified common barriers that researchers in LMICs face on local, national, and international scales. Appreciation for the various and diverse obstacles facing LMIC investigators marks a critical step in developing strategies toward creating equitable partnerships and, thus, decolonizing global health. Several organizations, including the Gates Foundation, have made efforts to decolonize global health by basing foundation representatives in LMICs. Further efforts should include the development of (1) global health centers in LMICs offering protected research time to LMIC investigators, (2) mentorship programs through which LMIC investigators may gain additional research training and support, (3) funding designated for projects with principal investigators from LMICs, and (4) implicit bias training for global health decision-makers in HICs. Strategies also must consider the unexpected consequences resulting from a shift in the global health paradigm. For example, disruption of funding and oversight mechanisms may pose a risk of broad collapse of global health research. To avoid disruption, funding agencies, investigators, and oversight committees in LMICs and HICs must collaborate to adapt global health infrastructure to align with the new paradigm.

## Notes

**Peer Reviewed**

**First published online: **January 19, 2024.
